# The acutely injured acromioclavicular joint – which imaging modalities should be used for accurate diagnosis? A systematic review

**DOI:** 10.1186/s12891-017-1864-y

**Published:** 2017-12-08

**Authors:** Jonas Pogorzelski, Knut Beitzel, Francesco Ranuccio, Klaus Wörtler, Andreas B. Imhoff, Peter J. Millett, Sepp Braun

**Affiliations:** 1 Department of Orthopaedic Sports Medicine, Hospital Rechts der Isar, University of Munich, Ismaninger Street 22, 81675 Munich, Germany; 2 Orthopaedic Surgery, The Steadman Clinic, Vail, CO USA; 3 Department of Radiology, Hospital Rechts der Isar, University of Munich, Munich, Germany

## Abstract

**Background:**

The management of acute acromioclavicular (AC) joint injuries depends on the degree of injury diagnosed by the Rockwood classification. Inadequate imaging and not selecting the most helpful imaging protocols can often lead to incorrect diagnosis of the injury. A consensus on a diagnostic imaging protocol for acute AC joint injuries does not currently exist. Therefore we conducted a systematic review of the literature considering three diagnostic parameters for patients with acromioclavicular (AC) joint injuries: 1) Assessment of vertical instability; 2) Assessment of horizontal instability; 3) Benefit of weighted panoramic views.

**Methods:**

Internet databases were searched in March 2016 using the terms (“AC joint” OR “acromioclavicular joint”) AND (MRI OR MR OR radiograph OR X-ray OR Xray OR ultrasound OR “computer tomography” OR “computed tomography” OR CT). Diagnostic, prospective, retrospective, cohort and cross- sectional studies were included to compare their use of different radiological methods. Case reports, cadaveric studies, and studies concerning chronic AC injuries and clinical outcomes were excluded.

**Results:**

This search returned 1359 citations of which 1151 were excluded based on title, 116 based on abstract and 75 based on manuscript. 17 studies were included for review and were analyzed for their contributions to the three parameters of interest mentioned above. The inter- and intra-observer reliability for diagnosing vertical instabilities of the clavicle using x-ray alone show a high level of reproducibility while for horizontal instabilities the values were much more variable. In general, digitally measured parameters seem to be more precise and reliable between investigators than visual classification alone. Currently, evidence for the value of weighted views and other additional diagnostic imaging to supplement standard x-rays is controversial.

**Conclusion:**

To date there is no consensus on a gold standard for diagnostic measures needed to classify acute AC joint injuries. The inter- and intra-observer reliability for diagnosing vertical instabilities of the clavicle using bilateral projections show a high level of reproducibility while for horizontal instabilities the results are much more inconsistent. There is currently no clear consensus on a protocol for image-based diagnosis and classification of acute AC joint injuries, leading to a lack of confidence in reproducibility and reliability.

## Background

Acute injuries of the AC joint are currently treated based on the grade of instability according to Rockwood’s classification. This classification was presented by Rockwood [1] in 1998 and is still widely accepted. The higher the Rockwood grade, the higher the severity and extent of injury of surrounding anatomical structures (Table 1).

**Table 1:** Rockwood classification system for AC joint injuries

Rockwood classification	Type I	Type II	Type III	Type IV	Type V	Type VI
AC ligaments	sprained	disrupted	disrupted	disrupted	disrupted	disrupted
CC ligaments	intact	sprained	disrupted	disrupted	disrupted	disrupted
Deltoid and trapezius muscle	intact	intact	detached from clavicle	detached from clavicle	detached from clavicle	detached from clavicle
Dislocation of the clavicle	none	AC joint widening, CC subluxation	AC joint dislocation, CC interspace 25–100% greater than compared to uninjured side	CC interspace may appear widened, clavicle horizontal unstable with posterior (sub-) luxation	AC joint dislocation, CC interspace more than 100% greater than compared to uninjured side	AC joint dislocation, clavicle displaced inferior to the coracoid

Successful management of AC joint instability is challenging for many reasons. A variety of non-anatomical and anatomical operative techniques is described, but there is no consensus which should be preferred. Moreover, the distinction between type III and IV injuries is still controversial. A solution to clarify this distinction would streamline treatment choices and may thus lead to more favorable clinical outcomes. As such, it is necessary to determine both vertical and horizontal instability with precise and reproducible methods. Radiographs are commonly used as a routine imaging tool in the assessment of shoulder injuries as they are widely available and provide convincing results [2, 3].

However, the literature suggests a broad range of imaging modalities and techniques that can be used to elucidate the extent of the AC joint disruption, including special X-ray images: alternative planes (e.g. bilateral Zanca [4], axillary view, dynamic axillary view), stress imaging, dynamic measures, indices, or additional modalities like ultrasound or MRI. Currently, a standardized protocol to image acute AC joint separations does not exist, rendering proficient and unanimous diagnosis difficult. The purpose of this review was to summarize all relevant available studies dealing with diagnostic imaging of acute AC joint injuries. A focus was set on three questions: 1) Assessment of vertical instability; 2) Assessment of horizontal instability; 3) Importance of weighted panoramic views.

## Methods

### Inclusion criteria

This systematic review was structured according to the PRISMA Checklist [5]. A literature search was performed focusing on studies reporting diagnostic imaging of acute AC joint injuries (diagnostic imaging within 3 weeks after injury) [6]. We included diagnostic, prospective, retrospective, cohort and cross- sectional studies comparing radiological methods or different applications of the same diagnostic technique. Publications written in English, German and Italian were all included.

### Exclusion criteria

We excluded case reports, cadaveric studies and studies on chronic AC injuries and clinical outcomes.

### Search strategy

Electronic databases (MEDLINE, COCHRANE Library, and EMBASE) were independently searched by two reviewers (*blinded for review*) on March 15, 2016 using the following search string: (“ac joint” OR “acromioclavicular joint”) AND (mri OR mr OR radiograph OR x-ray OR xray OR view OR ultrasound OR “computer tomography” OR “computed tomography” OR ct). In addition, the references of the included fulltext articles and available review articles were searched for additional studies that met the inclusion criteria.

### Study selection

In order to include and exclude studies according to the criteria above, two reviewers (*blinded for review*) searched the titles and abstracts of all identified publications and the fulltext of the eligible articles. Divergences were resolved in collaboration with a third reviewer (*blinded for review*).

### Data extraction

The data from all included articles were analyzed by two independent reviewers (*blinded for review*). Studies meeting all eligibility criteria were reviewed and the following data were abstracted: study design, level of evidence, type of imaging, number of patients, classification adopted and results. Level of evidence was assigned according to the widely accepted common grading of Evidence Levels for Primary Research. The abstracted data for each study were sorted into one of the following 3 categories: 1) Assessment of vertical instability; 2) Assessment of horizontal instability; 3) Requirement of weighted panoramic views.

## Results

**Fig. 1:**
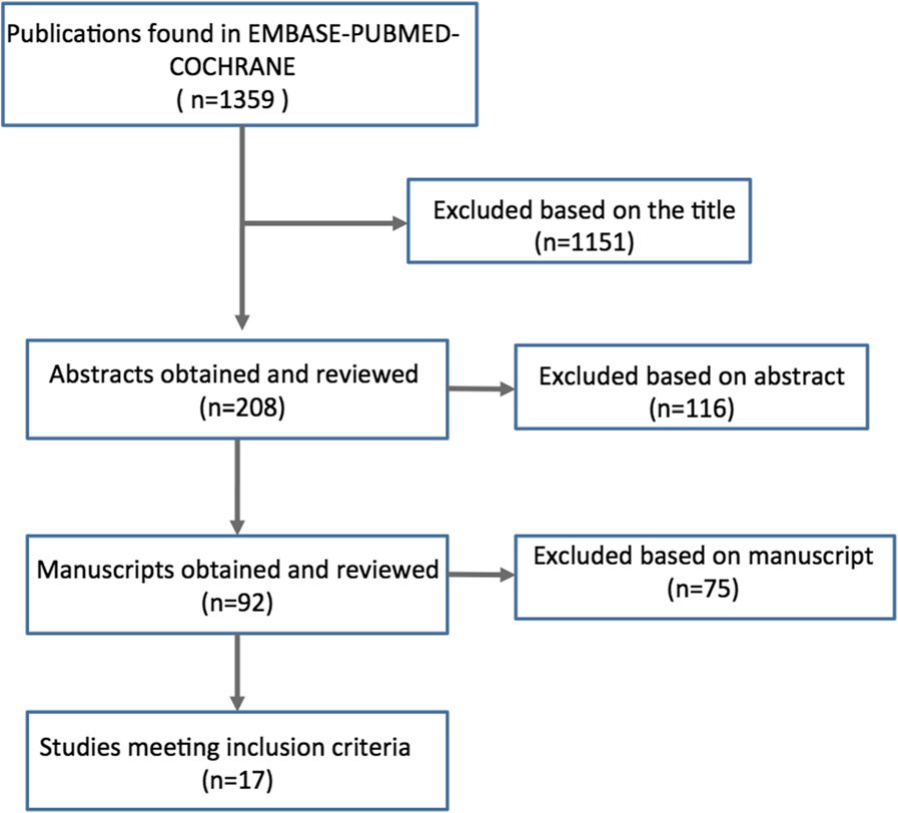
Flowchart of the study selection process

Figure 1 demonstrates the results of the literature search. 17 out of 1359 studies were extracted according to the inclusion criteria and are presented in Tables 2, 3 and 4.

**Table 2:** Assessment of vertical instability

Authors	Year of publication	Study design/level of evidence	Number of patients	Classification	Type of imaging	Results (according to classification of Landis/Koch [33])
Kraeutler et al. [34]	2012	Diagnostic study/IV	28	Rockwood	Anteroposterior and axial views: visual classification	Visual (ICC out of 8 investigators): IeOR = moderate (0.60) IaOR = good (0.69)
Cho et al. [15]	2014	Diagnostic study/I	28	Rockwood	Bilateral plain anteroposterior and axial views: visual classification. Bilateral plain anteroposterior and axial views and 3D–CT: visual classification.	Bilateral plain anteroposterior and axial views: visual (ICC out of 10 investigators): IeOR = slight (0.21) IaOR = moderate (0.47) Bilateral plain anteroposterior and axial views and 3D–CT: visual (ICC out of 10 investigators): IeOR = poor (0.18) IaOR = moderate (0.57)
Schneider et al. [7]	2014	Retrospective case series/IV	58	Rockwood	Bilateral panoramic stress and axial views: visual and digitally measured (CCI and HD) classification	Visual (range out of 4 investigators): IeOR = good (0.72–0.74) IaOR = good to excellent (0.67–0.93) Digitally measured CCI: IeOR = excellent (0.85–0.93) IaOR = excellent (0.90–0.97)
Gastaud et al. [9]	2015	Diagnostic study/I	15	Rockwood	Bilateral comparative anteroposterior views (Zanca-view [4]), axial views and dynamic axial views (Tauber [8]-protocol): digitally measured (CCI, D/A-ratio, X/Y-ratio, GACA) classification	Digitally measured CCI (range out of 4 investigators): IeOR = good to excellent (0.69–0.92) IaOR = good (0.60–0.77) Digitally measured D/A-ratio: IeOR = good (0.62–0.67) IaOR = good to excellent (0.66–0.95)
Schmid and Schmid [23]	1988	Diagnostic study/IV	22	Tossy	Intra-operative findings vs. ultrasound	• Only Tossy III: 100% accordance in classification
Fenkl and Gotzen [20]	1992	Diagnostic study/IV	35	Tossy	Weighted x-ray vs. ultrasound	• 97.2% accordance in classification • 2.8% underestimation of Tossy grade in ultrasound
Matter et al. [16]	1995	Diagnostic study/IV	20	Tossy	Weighted x-ray vs. ultrasound	• Ultrasound: Tossy III: 8.8 ± 2.1 mm AC-distance • Weighted x-ray: Tossy III: 8.4 ± 3.7 mm AC-distance
Kock et al. [35]	1996	Diagnostic study/IV	29	Tossy	Weighted x-ray vs. ultrasound	• Ultrasound: Tossy II: 0.48 ± 0.18 AC-Index Tossy III: 0.20 ± 0.03 AC-Index • Weighted x-ray: Tossy II: 0.50 ± 0.19 AC-Index Tossy III: 0.20 ± 0.03 AC-Index
Iovane et al. [22]	2004	Diagnostic study/IV	18	Rockwood	Weighted x-ray vs. ultrasound	• 100% accordance in classification (only Rockwood I – III)
Schaefer et al. [3]	2006	Diagnostic study/IV	13	Rockwood	Non-weighted x-ray vs. mri	• 84.6% accordance in classification • 15.4% underestimation of Rockwood grade in x-ray
Takase [18]	2011	Diagnostic study/IV	25	Rockwood	Non-weighted x-ray vs. mri	• 92% accordance in classification • 8% overestimation of Rockwood grade in x-ray
Nemec et al. [19]	2011	Diagnostic study/IV	44	Rockwood	Non-weighted x-ray vs. mri	• 52.2% accordance in classification • 36.4% overestimation of Rockwood grade in x-ray • 11.4% underestimation of Rockwood grade in x-ray

ICC = intraclass correlation coefficient, IeOR = inter-observer reliability, IaOR = intraobserver Reliability, CCI = coracoclavicular index, AC-width index = acromioclavicular-width index [10], D/A-ratio = vertical displacement of the clavicle^9^

**Table 3:** Assessment of horizontal instability

Authors	Year of publication	Study design/level of evidence	Number of patients	Classification	Type of imaging	Results (according to classification of Landis / Koch [33])
Schneider et al. [7]	2014	Retrospective case series/IV	58	Rockwood	Bilateral panoramic stress and axial views: visual and digitally measured (CCI and HD) classification	Digitally measured HD: IeOR = good to excellent (0.62–0.96) IaOR = good to excellent (0.67–0.98)
Vaisman et al. [10]	2014	Diagnostic study/II	40	Rockwood	Introduction of the AC-width index	Width index of ≥60%: • sensitivity of 95.7% and specificity of 97.5% • positive predictive value of 96.7% and negative predictive value of 95.6% for detecting a Rockwood grade IV injury
Tauber et al. [8]	2010	Diagnostic study/II	25	Rockwood	Introduction of the GACA	Cutoff value of 12.3°: • sensitivity of 93% and specificity of 92% • true-negative in 92% and false-negative in 8% for detecting a Rockwood grade IV injury
Gastaud et al. [9]	2015	Diagnostic study/I	15	Rockwood	Bilateral comparative anteroposterior views (Zanca-view^4^), axial views and dynamic axial views (Tauber^8^-protocol): digitally measured (CCI, D/A-ratio, X/Y-ratio, GACA) classification	Digitally measured X/Y-ratio: IeOR = moderate to good (0.48–0.80) IaOR = moderate to good (0.49–0.72) Digitally measured GACA: IeOR = poor to fair (0.01–0.33) IaOR = poor to fair (0.09–0.38)

AC-width index = acromioclavicular-width index^10^, GACA = gleno-acromio-clavicular-angle^8^, IeOR = inter-observer reliability, IaOR = intraobserver Reliability, HD = horizontal dislocation

**Table 4:** Requirement of weighted panoramic views

Authors	Year of publication	Study design/level of evidence	Number of patients	Classification	Kind of imaging? Pro weighted or pro non-weighted?	Justification
Bossart et al. [11]	1988	Diagnostic study/IV	83	Tossy	Weighted vs. non-weighted bilateral comparative x-rays. Pro non-weighted.	„use of weighted radiographs lacks efficacy in unmasking grade III AC sprains on radiograph “ [11]
Ibrahim et al. [12]	2015	Retrospective case series/IV	59	Rockwood	Weighted vs. non-weighted bilateral comparative x-rays. Pro weighted.	“value of bilateral weighted views is to ‘unmask’ a grade V injury“ [12]
Izadpanah et al. [13]	2013	Diagnostic study/IV	10	Rockwood	Non-weighted x-ray vs. weighted x-ray and non-weighted mri vs. weighted mri Pro weighted.	“application of stress (...) enables a partial rupture to be dis-tinguished from a complete ligamental rupture“ [13]

All 17 eligible studies were scrutinized three times, each time pertaining to each of the 3 previously asked questions. The results of each question will be discussed separately below.

### Assessment of vertical instability

Twelve out of the 17 studies revealed results specifically pertaining to assessment of vertical instability. Of interest in each study were the levels of inter-observer reliability, intra-observer reliability and the preferred diagnostic method. As a general result, the inter- and intra-observer reliability for diagnosing vertical instabilities of the clavicle showed a high level of reproducibility with mostly good to excellent results. In addition, digitally measured results showed a trend towards superior results compared to those of visually evaluated x-rays. Although radiographs and ultrasound obtained almost identical values and showed comparable measurement accuracy, x-ray remains mandatory to exclude fractures and is examiner-independent. This is why radiographs appear to be superior compared to ultrasound in the diagnosis of the acutely injured AC joint. Further relevant information is presented in Table 2.

### Assessment of horizontal instability

Four out of the 17 studies revealed results specifically pertaining to assessment of horizontal instability at the AC joint. Schneider et al. examined the inter- and intra-observer reliability of visual evaluation, which showed mostly good results, and also digitally measured horizontal displacement of the clavicle, which demonstrated superior good to excellent results [7]. Tauber et al. devised the gleno-acromio-clavicular-angle (GACA) to quantify the horizontal clavicular dynamics measured on X-rays [8]. With the help of dynamic views they were able to reveal horizontal instability in six patients, which had previously been classified as “stable” on static axillary views. Gastaud et al. also assessed gleno-acromio-clavicular-angle (GACA) [8] as well as the X/Y-ratio (horizontal displacement of the clavicle), which showed extremely variable inter- and intra-observer reliability from poor to good [9]. Additionally, Vaisman et al. devised the AC-width index (the difference between the AC-width on the injured side and the normal side, subsequently divided by the AC width of the normal side) and suggested an AC-width index value ≥0.6 to be highly predictive of a horizontally unstable AC joint [10]. Further relevant information is given in Table 3.

### Requirement of weighted panoramic views

Three out of the 17 studies revealed results discussing the utility of weighted panoramic views. Bossart et al. questioned the routine use of stress views for acutely injured AC joints as they compared weighted and non-weighted views of acutely injured AC joints and could only detect in 3 out of 84 cases (4%) an unmasked Tossy III injury with the help of weighted films [11]. Ibrahim et al. suggested weighted views may be useful because of their ability to uncover Rockwood V injuries [12]. This study reported 10 out of 59 (17%) patient diagnoses increased to Rockwood V injuries when weighted films were used. This was further validated by Izadpanah et al. using MRI with and without stress [13]. In all 10 cases they reported, a precise outline of the CC-ligaments and a differentiation between sprained and torn ligaments could be obtained, suggesting weighted MRI provides a significant diagnostic advantage. Further relevant information is given in Table 4.

## Discussion

Our review revealed that only 17 studies, with a low level of evidence varying between level II-IV, met our inclusion criteria. Bilateral panoramic view with digitally measured CC-distance allows for the most accurate diagnosis of vertical instability (type V instability). For the identification of horizontal instability (as seen in type IV instability), data is still insufficient with variable results and multiple diagnostic methods discussed. Due to the heterogeneity of the published literature, no gold standard for the imaging of the AC joint instability can be presented. However, basic principles in the utilized imaging modalities could be identified. In general, radiographs seem to be the preferred diagnostic method due to their wide availability and examiner-independence, which separates it from ultrasound. MRI and CT imaging generally play a subordinate role in the diagnosis of the acutely injured AC joint, mainly due to high costs and poor availability. Moreover, in cases of acute AC joint injuries, the use of MRI and CT might not add additional significant information, as there is normally no need to evaluate bones, nerves or vessels in detail [14].

### Assessment of vertical instability

The vertical displacement of the clavicle is found to be reproducible showing strong concordance for intra- and inter-observer reliability. It should be diagnosed as CC-distance rated on bilateral panoramic views. This may be because the coracoid process and the acromion can be easily identified on x-ray and serve as reliable reference points. Since the classification of Rockwood is based on the relative increase of the CC distance compared to the contralateral side, panoramic views allow direct correlation to the uninjured contralateral joint. Results measured digitally seem to be more accurate than those taken visually [7, 9, 15]. One interpretation could be that digitally measuring the extent of the injury is more objective and can be performed with a systematic approach if predetermined diagnostic parameters exist, however visual diagnosis is subjectively based on the experience of the physician.

Several authors have tested alternative imaging modalities such as MRI, ultrasound and computer tomography versus X-ray [3, 13, 15, 16]. The literature suggests that MRI is able to capture superior detail – allowing it to differentiate a sprain from torn ligaments and fascial injury, as well as revealing concomitant intraarticular pathologies of the glenohumeral joint which occur up to 18% of the time [17]. In 2 out of 3 studies, there was a high level of concordance between the results from MRI and X-ray [3, 18]. Only Nemec et al. reported a lack of concordance in Rockwood classification results between MRI and X-ray [19]. However, this may be explained by their patients mainly being graded Rockwood II and III where MRI is most useful because of direct visualization of the AC- and CC-ligaments. Since the patient is examined in the supine position, the weight of the arm does not affect the CC-distance and the scapula will not be able to go into the protracted position, widely encountered in AC-joint instabilities. These factors should also be taken into account, when evaluation MRI in the presentation of an acute AC-joint injury. This mechanical effect is more relevant, if the injury is not acute. In these cases, MRI might demonstrate a ligamentous structure with a structural continuity but without adequate tension of the ligaments.

Ultrasound has been shown to be able to reliably differentiate between sprain and rupture of the AC- and CC-ligaments and is widely available and inexpensive [16, 20, 21]. Therefore it is an interesting addition to the current diagnostic work up which is mainly based on conventional X-ray. Studies evaluating the use of ultrasound for diagnosing AC joint lesions showed results of very good concordance with X-ray and intra-operative findings [20, 22, 23]. The advantage of sonographic evaluation is rather seen in the detection of horizontal instability with the advantage of functional testing to demonstrate the overriding of the lateral clavicle over the acromion. However, ultrasound still provides a rather subjective result and highly depends on the experience of the user. The advantage in conventional X-ray imaging is seen in the possibility to exclude fractures and the direct correlation to Rockwood’s criteria.

Using CT, Cho et al. compared intra- and inter-observer reliability, diagnosing acute AC joint injuries with X-ray alone and X-ray augmented with 3D CT [15]. Although the addition of 3D CT improved reliability, it did not reach statistical significance. With the associated radiation exposure and cost, the routine use of 3D CT in clinical practice again may not be justified.

### Assessment of horizontal instability

Correct and reliable diagnosis of a horizontal instability is important since the discrimination of a type III injury vs. a type IV injury determines whether the basic treatment recommendation is surgical or non-surgical. It is widely accepted that a horizontally unstable clavicle requires operative management, meaning correct diagnosis of Rockwood IV injuries is imperative [24]. Unfortunately, this diagnosis is often difficult because in most cases, the diagnose of a mainly 3D dynamic pathology has to be made based on a static imaging modality.

Radiographs taken in the axillary projection have been a standard modality to diagnose horizontal instability in the past. The patient is in a supine position (scapula fixed – no effect of bodyweight or scapula position) and the correct projection for good quality requirescompliance of the patient as well an experienced technician. Rahm et al. showed in a cadaver model that using a standard axillary radiograph to diagnose horizontal instability has a very high sensitivity but low specificity, where a variation in axillary views can misguide the examiner to interpret X-Rays to have posterior clavicular translation [25]. Additionally, they found that a small variation in the beam angle has a large effect on measurements taken because of distorted images. Moreover, Barth et al. could show recently that the uninjured AC joint is not perfectly aligned anteriorly and posteriorly in as much as 40% of the cases [26]. This finding might be another explanation for the poor results when interpreting horizontal instability on imaging. As a solution, Vaisman et al. used the AC-width index, a method to detect instability of the clavicle without the need of an axillary view [10]. Even though they presented superior results, there is no further study which could confirm these findings at the present time. Tauber et al. pointed out that a standing or sitting position when taking an axillary view results in dropping the affected shoulder, which subsequently malrotates the scapula, which could potentially conceal the extent of the lesion [8]. They referred to Alexander [27] who described a modification of the axillary view in 1949 with the patient sitting or standing and the shoulders thrusted forward at the time when the x-ray is taken. Thus they recommended removing the vertical forces by taking axillary views in a supine position. They performed a dynamic examination with two lateral views with the arm at 90° of abduction and an additional 60° of flexion or extension to evaluate the horizontal dynamics of the lateral clavicle. The GACA was measured and used to quantify the horizontal instability of the clavicle in terms of angle differences. Although the authors could present very good values for intra- and inter-observer reliability with a sensitivity of 93% and a specificity of 92%, Gastaud et al. could not confirm these results in a study presented in 2015 concluding “that the horizontal displacement was difficult to evaluate on axillary lateral views and that the dynamic instability could not be reproducibly and reliably evaluated” [9]. An explanation may be that lateral axillary views contours overlap, and thus anatomical landmarks could be misinterpreted.

A projection of an axillary X-ray first described by Alexander [27] in 1949 has only been mentioned in the literature, but no study demonstrating its sensitivity or specificity has been published until now [27, 28]. The advantage of this projection is the application of “stress” on the AC joint through the flexion/adduction of the arm. It has been widely suggested by clinicians with increased interest in the last decade, but there is a lack of supporting evidence of their advantage over other views.

### Diagnostic value of weighted panoramic views

For decades, surgeons all over the world have debated whether weighted or non-weighted views are more effective in correctly diagnosing AC joint injuries. According to one theory, pain triggered muscle spasm may “mask” the total extent of an injury, making weighted films necessary in order to distract the AC joint [29, 30]. In addition, some authors recommended that the weights should not be held in the hand, but suspended from the wrists to minimize voluntary muscle contraction [31]. Despite no clinical study validating this theory, it remained widely accepted for a long time. The first study challenging the requirement of weighted views was Bossart et al. in 1988 [11]. Using a clinical trial, they showed that in the vast majority of AC joint injuries, weighted films did not change the grade of injury. Due to low diagnostic yield and additional patient discomfort, they recommended weighted views for acute AC joint injuries should be abandoned. This opinion prevailed in the upcoming years, and the use of weighted radiographs became less popular. Yap et al. published a survey showing that a large majority of the specialized American shoulder and elbow community did not use weighted views in daily practice [32]. However, recently, Izadpanah et al. and Ibrahim et al. published studies indicating that stress views provide additional information helping to guide management [12, 13]. Both authors showed a significant increase in the CC distance when weights were applied, sometimes resulting in an upgrading of Rockwood III injuries, potentially changing therapy from conservative to operative treatment. In summary, because so few studies have reported on this topic, it is not possible to confidently advocate whether or not weighted views should be used routinely.

A fundamental problem encountered in most included studies is that the true extent of the injury can only be defined upon assessing the injury intra-operatively. Therefore, when comparing imaging results alone without any intraoperative reference, the assessor can never reliably assess the accuracy of an imaging method. Additionally, by only assessing articles evaluating acute AC joint injuries, following our exclusion criteria, our findings may not be able to be extrapolated to chronic AC joint injuries. Due to this significant lack of evidence, no gold standard for the imaging of AC joint instability can be defined. However, further development of optimal treatment strategies requires a reflected application of a set of all modalities to optimize the information and make the diagnosis as precise as possible. In the authors’ current practice, panoramic views in combination with axial and or “Alexander” projections are used. Additional imaging such as MRI or CT is added if further information is required. In addition, current publications try to combine the advantages of clinical examination and imaging through a combined decision-making process. If the patient presents with a high level of pain in the acute phase following trauma, a second evaluation after some days might allow a better diagnose due to less protective muscle activation of the patient [24].

This systematic review has several limitations. First, the partial low level of evidence of the included studies predetermines the level of evidence of our study. Secondly, most of the included studies contain only small patient numbers. Thirdly, some of the publications were based on the Tossy classification, which has largely become obsolete. Despite this, many of these studies provided valuable information justifying their utilization.

## Conclusion

A gold standard to investigate and diagnose acute AC joint injuries does not currently exist. The inter- and intra-observer reliability for diagnosing vertical instabilities of the clavicle using bilateral projections show a high level of reproducibility while for horizontal instabilities the results are much more heterogeneous. Currently, the evidence is conflicting over whether weighted views and additional diagnostics can usefully augment results from standard X-ray views. There is currently no consensus on a protocol to view acute AC joint injuries; therefore, classifying these injuries with high levels of confidence, reproducibility and reliability remains difficult. To improve clinical outcomes it may be of high importance to precisely diagnose AC injuries, which asks for an easily feasible, reproducible and safe diagnostic measure.

## Abbreviations

AC: Acromioclavicular
CC: Coracoclavicular
CCI: Coracoclavicular index
CT: Computed tomography
D/A-ratio: Vertical displacement of the clavicle
GACA: Gleno-acromio-clavicular angle
MRI: Magnetic resonance imaging
